# Inactivation and spike protein denaturation of novel coronavirus variants by Cu_*x*_O/TiO_2_ nano-photocatalysts

**DOI:** 10.1038/s41598-023-30690-0

**Published:** 2023-03-10

**Authors:** Tetsu Tatsuma, Makoto Nakakido, Takeshi Ichinohe, Yoshinori Kuroiwa, Kengo Tomioka, Chang Liu, Nobuhiro Miyamae, Tatsuya Onuki, Kouhei Tsumoto, Kazuhito Hashimoto, Toru Wakihara

**Affiliations:** 1 School of Engineering, The University of Tokyo, 7-3-1, Hongo, Bunkyo-Ku, Tokyo, 113-8656 Japan; 2 Institute of Industrial Science, The University of Tokyo, 4-6-1 Komaba, Meguro-Ku, Tokyo, 153-8505 Japan; 3 Institute of Medical Science, The University of Tokyo, 4-6-1 Shirokanedai, Minato-Ku, Tokyo, 108-8639 Japan; 4 Nippon Paint Co., Ltd, 4-1-15 Minamishinagawa, Shinagawa-Ku, Tokyo, 140-8675 Japan

**Keywords:** Nanoparticles, Photocatalysis, Biochemistry

## Abstract

In order to reduce infection risk of novel coronavirus (SARS-CoV-2), we developed nano-photocatalysts with nanoscale rutile TiO_2_ (4–8 nm) and Cu_*x*_O (1–2 nm or less). Their extraordinarily small size leads to high dispersity and good optical transparency, besides large active surface area. Those photocatalysts can be applied to white and translucent latex paints. Although Cu_2_O clusters involved in the paint coating undergo gradual aerobic oxidation in the dark, the oxidized clusters are re-reduced under > 380 nm light. The paint coating inactivated the original and alpha variant of novel coronavirus under irradiation with fluorescent light for 3 h. The photocatalysts greatly suppressed binding ability of the receptor binding domain (RBD) of coronavirus (the original, alpha and delta variants) spike protein to the receptor of human cells. The coating also exhibited antivirus effects on influenza A virus, feline calicivirus, bacteriophage Qβ and bacteriophage M13. The photocatalysts would be applied to practical coatings and lower the risk of coronavirus infection via solid surfaces.

## Introduction

Coronavirus disease 2019 (COVID-19) was first reported as a pneumonia of unknown cause in December 2019, and its pathogen was identified as a novel coronavirus (SARS-CoV-2) in January 2020. The disease caused an outbreak, and the World Health Organization (WHO) declared a pandemic in March 2020. The coronavirus is characterized by its strong infectivity. Some of the mutation variants are known to be more infectious, and were classified as variants of concern. The most major pathway of the COVID-19 transmission is believed to be airborne aerosol including viruses released from infected persons. However, there are also pathways via solid surfaces including walls, doorknobs, handrails and furniture^1^. Removal of viruses from the surfaces would therefore reduce the risk of transmission of the disease. Photocatalyst is one of the promising materials for virus removal. A decade ago, Hashimoto and co-workers^2,3^ reported that TiO_2_ photocatalysts modified with Cu_*x*_O inactivate bacteriophage Qβ. The Cu_*x*_O/TiO_2_ composites absorb visible light and electrons are excited from the TiO_2_ valence band (VB) to Cu(II), and Cu(II) is reduced to Cu(I), which inactivates bacteriophage. The positive holes generated in the TiO_2_ VB take electrons from ambient water, so that TiO_2_ is initialized. Although Cu(I) in Cu_*x*_O is ready to be oxidized back to Cu(II) by ambient oxygen, the Cu_*x*_O with Cu(I) is renewed under illumination on the basis of the photocatalytic effect mentioned above.

With these mechanisms in mind, we developed novel photocatalysts that inactivate the novel coronavirus and its variant, as well as some other viruses. White and translucent paint coatings containing those photocatalysts were also developed. In the previous work, a rutile TiO_2_ powder of 0.1 − 0.3 µm or larger in size was used as a typical base semiconductor material, and Cu_*x*_O nanoparticles of ~ 5 − 8 nm diameter were deposited onto the TiO_2_ powder^2,3^. Considering its large composite size and high refractive index of rutile TiO_2_ of 2.5 − 3.0 in the visible wavelength range^4^, the composite particles should tend to settle in a dispersion^5^ and reflect or scatter visible light greatly.

In the present work, we employed a sol containing rutile TiO_2_ nanoparticles of ~ 4 − 8 nm in size, and deposited Cu_*x*_O clusters of < 2 nm in size to develop photocatalysts with a high active surface area. In addition, because of the nanoscale particle size, we can fabricate translucent coatings, films and solid substrates containing the photocatalysts, which possess high designability and allow one to irradiate them both from the frontside and backside. An additional advantage of the small nanoparticles is high suspendability without sedimentation, because sedimentation velocity of nanoparticles smaller than 10 − 100 nm is negligibly low^5^. This is important point because only photocatalysts exposed at the coating surface are expected to affect viruses. The present sol-based wet process for preparing nano-photocatalyst coatings would allow various types of antivirus materials including paints, varnishes, gels and spray liquids to be developed. A sol containing anatase TiO_2_ nanoparticles (~ 10 nm) was also used in the present study in place of the rutile TiO_2_ sol. We found that the nano-photocatalyst coatings renew Cu_*x*_O under visible light illumination and inactivate a variety of viruses including the original and alpha variants of novel coronavirus. In order to elucidate the inactivation mechanisms, we prepared recombinant proteins of the receptor binding domain (RBD) of spike protein derived from the original, alpha and delta variants. The photocatalysts greatly suppressed binding ability of RBD to human angiotensin converting enzyme-2 (ACE2), the receptor for the coronavirus.

## Methods

### Preparation of Cu_*x*_O/TiO_2_ nanocomposites

A rutile TiO_2_ slurry (Tayca TS-310, TiO_2_ diameter ~ 4 − 8 nm) and an anatase TiO_2_ slurry (Taki Chemical M-6, TiO_2_ diameter ~ 10 nm) were employed as base semiconductor materials, and aqueous solutions of CuCl_2_, glucose as a reducing agent and NaOH were added to either of the slurries. The mol ratio of Cu to Ti was 1/20, unless otherwise noted (concentrations: 437 or 404 mM TiO_2_; 62 or 57 mM glucose; 47 or 48 mM NaOH; 22 or 20 mM CuCl_2_ for rutile and anatase, respectively). We raised its temperature to 90 °C and stirred it for 1 h to reduce Cu(II) ions to Cu(I) and deposit Cu_*x*_O, which is a mixture of Cu_2_O and CuO, onto TiO_2_ nanoparticles.

### Preparation of latex paints

A white pigment based on rutile TiO_2_ coated with Al_2_O_3_ and ZrO_2_ (CR-97, Ishihara Sangyo; ~ 400 nm diameter), which has no photocatalytic activity because it is coated with an inert layer, was employed as a typical pigment for practical paints. It was mixed with CaCO_3_, diatomite, wetting and dispersing additive (Disperbyk 190, BYK), defoamer (BYK-011, BYK) and water (weight ratio = 40:20:10:5:1:13). The slurry thus obtained was further mixed with acrylic emulsion (Watersol AC 3080, DIC), 2,2,4-trimethyl-1,3-pentanediolmonoisobutyrate and the Cu_*x*_O/TiO_2_ slurry (weight ratio = 40:1:20) to obtain a photocatalytic latex paint. For studies on antivirus effects, the paint was applied onto a glass plate (20 × 20 mm).

### Characterization

A solar simulator (AM1.5, ~ 100 mW cm^-2^; BSS-T150, Bunko Keiki) and fluorescent lamps for consumer use were used for light irradiation. Absorption spectra were collected by using a spectrophotometer V-670 (Jasco). Scanning transmission electron microscopy (STEM) and high resolution HAADF-STEM analysis were performed by using JEM-ARM200F Thermal FE STEM (JEOL). For energy dispersive X-ray spectroscopy (EDS), DRY SD100GV (JEOL) was used. Evaluation methods for antivirus activity are described in Results and discussion section.

### Expression and purification of recombinant proteins

As for RBD protein, gene fragments encoding RBD protein were cloned into pcDNA 3.4, an expression vector for mammalian expression system (Thermo Fisher Scientific) with a signal peptide sequence, His-tag, and TEV protease recognition sequences at the N-terminal end. Expi293 cells (Thermo Fisher Scientific) were transfected with the expression vector and supernatant was collected 4 days after transfection. The supernatant was dialyzed against a binding buffer consisting of 20 mM Tris–HCl (pH 8.0), 500 mM NaCl and 5 mM imidazole and loaded on Ni–NTA resin (Qiagen) equilibrated with the binding buffer. The resin was washed with a wash buffer consisting of 20 mM Tris–HCl (pH 8.0), 500 mM NaCl and 20 mM imidazole and subsequently RBD protein was eluted by a buffer consisting of 20 mM Tris–HCl (pH 8.0), 500 mM NaCl and 500 mM imidazole. The eluted RBD protein was dialyzed against binding buffer with TEV protease to cleave the His-tag, followed by loading on Ni–NTA resin. Flowthrough fraction was collected and further purified by size exclusion chromatography using HiLoad 26/600 Superdex 75-pg column (Cytiva) equilibrated with PBS. As for ACE2 protein, a gene fragment encoding ACE2 protein was also cloned into pcDNA 3.4 vector and used for transfection of Expi293 cells. The supernatant of infected cells was collected 5 days after transfection and ACE2 protein was purified using Ni–NTA and size exclusion chromatography in the same way as RBD protein. The monomer peak fractions were collected for each protein and the purity was evaluated by SDS-PAGE followed by Coomassie staining.

### Protein denaturation and ELISA assay

RBD proteins were incubated with a photocatalyst at 4 °C overnight. Subsequently, RBD proteins were immobilized on an ELISA plate (Corning) at 4 °C overnight. The protein immobilized wells were blocked by skimmilk containing PBS-T buffer and ACE2 proteins were added to each well and incubated at room temperature for 1 h. The wells were washed 3 times with PBS-T and bound ACE2 were detected with anti-His-tag antibody conjugated with HRP (MBL Life Science). The wells were washed 3 times with PBS-T and developed with TMB substrate mixture (Cosmobio) and stopped with TMB stop buffer (ScyTek Laboratories). The absorbance at 450 nm for each well was measured using Pherastar plate reader (BMG Labtech).

## Results and discussion

### Preparation and characterization of Cu_*x*_O/TiO_2_

**Figure 1:**
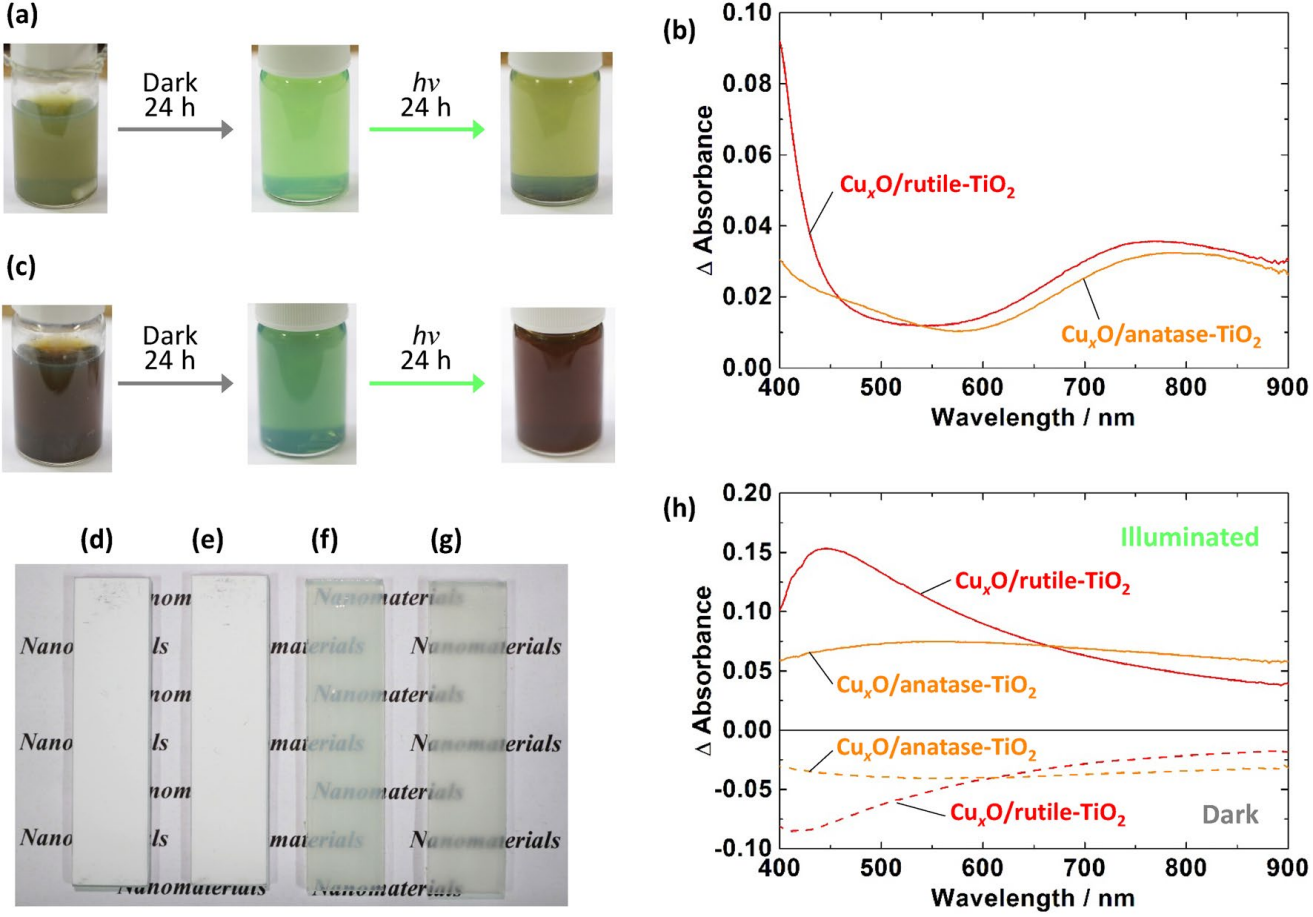
Colour and spectral changes of the photocatalysts. (**a**, **c**) Colour changes of the (**a**) rutile- and (**c**) anatase-based photocatalyst suspensions after leaving in the dark and under irradiation with simulated solar light. (**b**) Spectra of the as-prepared photocatalyst suspensions. (**d**–**g**) Photographs of the (**d**, **f**) rutile- and (**e**, **g**) anatase-based photocatalyst coating (**d**, **e**) with or (**f**, **g**) without white pigments. (**h**) Spectral changes of the rutile- and anatase-based coatings in the dark and under illumination (fluorescent light, > 380 nm, 500 lx).

The colour of the rutile-based Cu_*x*_O/TiO_2_ suspension was a greenish gray (Fig. 1a), and its difference spectrum after the deposition was characterized by an absorption band at 400 − 500 nm and a broad peak at ~ 800 nm (Fig. 1b). The latter broad peak suggests that the suspension contains excess Cu^2+^ ions. Since the former absorption band appears to be due to a semiconductor, we examined Tauc plots and obtained the band-gap values of ~ 3.0 and ~ 2.8 eV on the assumption of the direct and indirect transitions, respectively. Because Cu_2_O and CuO have been reported to have direct allowed transition band-gap of 2.1 − 2.6 eV and indirect allowed transition band-gap of 1.2 − 1.6 eV, respectively^6–10^, we conclude that the optical behaviour observed in the short wavelength range, from which absorption of TiO_2_ has been excluded, is attributed mainly to Cu_2_O. The wider band-gap in comparison with bulk Cu_2_O could be due to the quantum-size effect, as discussed later. Since the Cu_*x*_O contains Cu_2_O, it is expected to exhibit an antivirus effect.

The anatase-based suspension showed a brownish gray colour (Fig. 1c) and an absorption band at 400 − 600 nm (Fig. 1b). The corresponding Tauc plots show that the band-gap is ~ 2.9 eV for direct transition and ~ 1.7 or ~ 2.3 eV for indirect transition. The optical behaviour could therefore be ascribed to both Cu_2_O and CuO.

**Figure 2:**
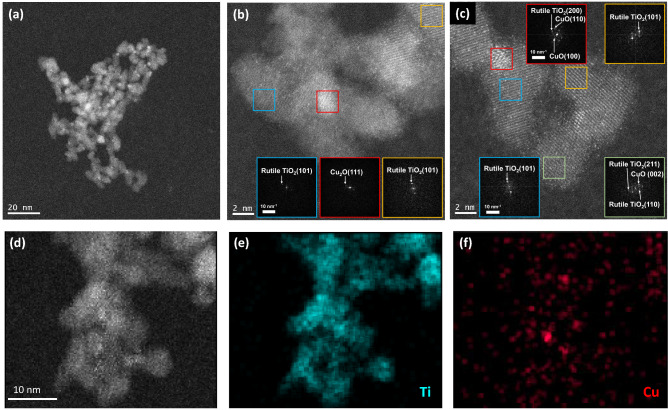
Photocatalyst nanoparticles. (**a**–**d**) HAADF-STEM images of the photocatalysts. (**e**, **f**) STEM-EDS elemental mapping images for (**e**) Ti and (**f**) Cu.

We also subjected the rutile-based suspension to STEM and EDS analyses after thorough evaporation of water from the suspension (Fig. 2). The STEM image (Fig. 2a) shows that the primary size of the nanoparticles is smaller than 10 nm. As a result of elemental mapping based on STEM-EDS analysis (Fig. 2d–f), we found that the major component was TiO_2_ nanoparticles of ~ 4 − 8 nm in size, and that clusters of Cu compounds (1 − 2 nm or less) were deposited on TiO_2_. High resolution HAADF-STEM analysis proved that the TiO_2_ nanoparticles were in rutile phase and that the Cu compound clusters contained both Cu_2_O and CuO (Fig. 2b, c). The quantum-sized Cu_2_O^11,12^ justifies its widened band-gap of ~ 3.0 eV mentioned above due to a quantum-size effect. Small particles generally give large specific surface area, high optical transmittance and good suspendability. Actually both rutile- and anatase-based suspensions showed no sedimentation for at least 1 year.

**Figure 3:**
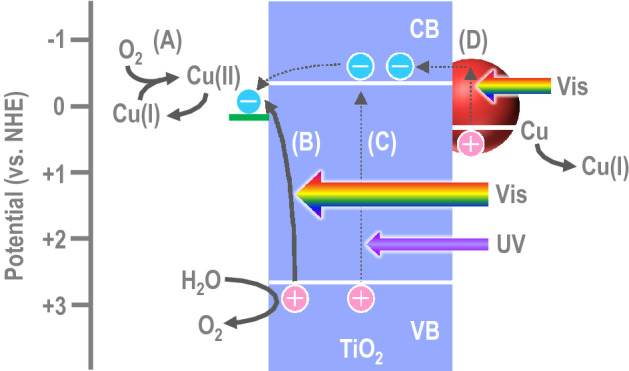
Photoinduced chemical processes involved in the present photo-renewable system. (A) Aerobic oxidation of Cu(I) to Cu(II). (B) Photo-induced interfacial charge transfer from the TiO_2_ VB to Cu(II). (C) Photo-excitation of electrons in the TiO_2_ VB to CB. (D) Plasmonic excitation of over-reduced, metallic Cu nanoparticles, which inject electrons to the TiO_2_ CB. Process B is the major photo-process and Processes C and D are minor processes.

When the suspensions were left in the dark, their colour was gradually changed to green in 24 h (Fig. 1a, c), suggesting that Cu_2_O was oxidized to Cu^2+^ by dissolved oxygen (Fig. 3, Process A). However, when we irradiated the oxidized suspensions with light from a solar simulator for 24 h, their colour was changed again to greenish gray (Fig. 1a) and brownish gray (Fig. 1c) for rutile- and anatase-based photocatalysts, respectively, indicating that Cu^2+^ was reduced back to Cu_2_O. This reduction can be explained in terms of photo-induced interfacial charge transfer from the TiO_2_ VB to Cu^2+^ at the TiO_2_ surface (Fig. 3, Process B)^2^ Resultant holes in the TiO_2_ VB should be consumed by oxidation of water to oxygen. Electrons in the TiO_2_ VB could also be excited to the conduction band (CB) under the simulated solar light, which contains weak UV light, and the excited electrons could also contribute to the reduction of Cu^2+^ (Fig. 3, Process C). In the case where the mol ratio of Cu to Ti was 1/100 or lower, a small absorption peak was observed at ~ 580 nm after irradiation of the anatase-based photocatalyst. This could be due to localized surface plasmon resonance (LSPR) of over-reduced, metallic Cu nanoparticles. Plasmonic metal nanoparticles in contact with TiO_2_ inject electrons to the TiO_2_ conduction band, and metal is oxidized to metal ions, in the case of Ag or less noble metals^13,14^ including Cu^15^ (Fig. 3, Process D).

### Photocatalytic coatings

Either (rutile or anatase) of the Cu_*x*_O/TiO_2_ suspensions was added to a latex paint containing inorganic white pigments and organic binders, followed by 5-min stirring and 1-min degassing. Each photocatalyst slurry thus obtained was applied onto a glass plate (0.01 mL cm^-2^). Solvent in the coating was evaporated at 25 °C for 7 days, and white films were obtained (Fig. 1d, e). Colourless translucent films without the white pigment (Fig. 1f, g) were also prepared for spectroscopic measurements. The small particle size of Cu_*x*_O/TiO_2_ is responsible for the good transmittance. Their average thickness was 50 µm for both photocatalyst films.

After preparation of the coatings, those were left in the dark. As a result, their absorption in the visible wavelength range was decreased gradually (Fig. 1h). In marked contrast, the absorption was gradually increased under irradiation with fluorescent light (< 380 nm light was cut off). The peak at 440 nm reflect photo-induced interfacial charge transfer from the TiO_2_ VB to Cu^2+^^16,17^. The absorption decrease in the dark and the increase under illumination can be explained in terms of aerobic oxidation (Fig. 3A) and photocatalytic re-reduction (Fig. 3B), respectively, of Cu_*x*_O clusters. Since Cu^+^ in Cu_*x*_O exhibits antivirus effects, the photocatalysts are expected to retain their antivirus activities, if any, under fluorescent light, even in the paint coatings.

### Antivirus effects of the coatings

**Figure 4:**
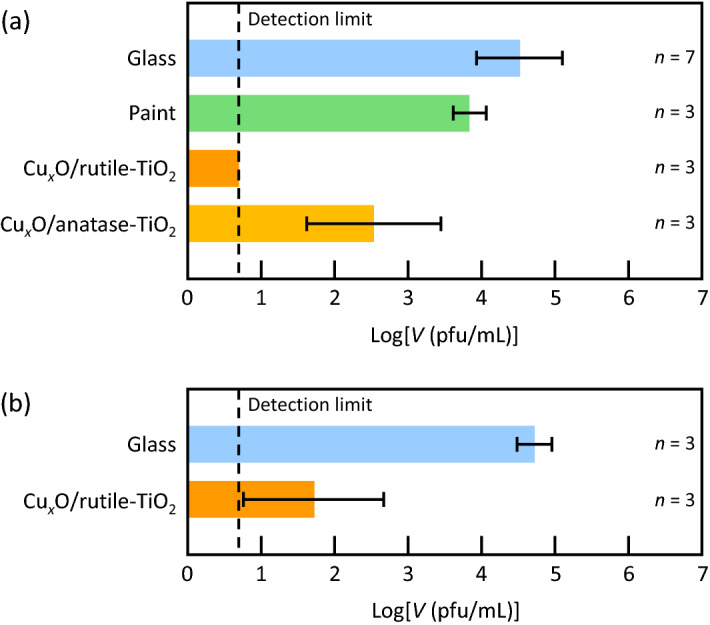
Antivirus effects of the photocatalyst coatings on novel coronaviruses. Viral infectivity (*V*) values for (**a**) the original and (**b**) alpha variants of novel coronavirus after incubation under fluorescent light (1000 lx for 3 h) are shown. Raw data are summarized in Table S1 in Supplementary Information.

The photocatalyst paint coatings were subjected to inactivation tests against novel coronavirus (SARS-CoV-2, original variant) according to the procedures given in International Organization for Standardization ISO 21,702 with some modifications. Coronaviruses in 5% FBS DMEM medium (25 µL) were applied onto a glass plate (20 × 20 mm) coated with the rutile- or anatase-based photocatalyst coating. The plate was covered with a polypropylene film of the same size and was incubated under fluorescent lamp illumination (1000 lx) for 3 h, followed by evaluation of viral infectivity *V* (in pfu mL^-1^) by a plaque assay. Figure 4a shows the log*V* values together with those for control experiments in which a bare glass plate or a glass plate coated with the paint without photocatalyst was used instead of the photocatalytic plate. The rutile-based coating strongly inactivated the novel coronavirus and the obtained viral infectivity was lower than the detection limit of 5 pfu. Its antivirus activity [= (log*V*)_ave_ – (log*V*_0_)_ave_, where *V*_0_ is viral infectivity for a bare glass plate and subscript ave stands for averaged values) is 3.8 or higher. The coronavirus was also inactivated by the anatase-based coating, whereas its antivirus activity was lower (2.0). This difference should be due to the lower interfacial charge transfer absorption band at 440 nm for the anatase-based photocatalyst in comparison with the rutile-based one (Fig. 1h). The larger band-gap of anatase TiO_2_ (3.2 eV) than rutile (3.0 eV), which lowers the contribution of the Cu^2+^ reduction pathway via the TiO_2_ CB (Fig. 3C), may also be responsible for the activity difference.

Next we examined an antivirus effect of the rutile-based coating on alpha variant (also known as lineage B.1.1.7 or VOC-202012/01) of the novel coronavirus, which has mutations including N501Y (Fig. 4b). Its antivirus activity was 3.0; the photocatalyst coating is also effective against alpha variant. Possible mechanisms of inactivation by the photocatalysts are discussed at the end of this section.

**Figure 5:**
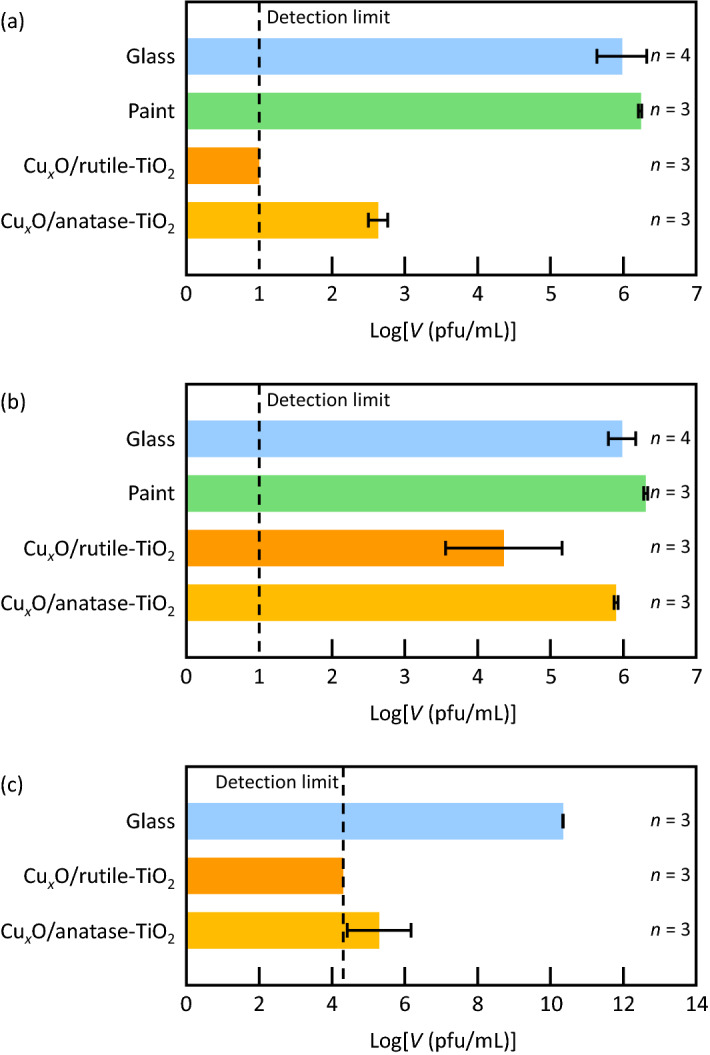
Antivirus effects of the photocatalyst coatings on bacteriophages. Viral infectivity (*V*) values for bacteriophage Qβ (**a**) after incubation under fluorescent light (500 lx for 4 h) or (**b**) in the dark and (**c**) those for bacteriophage M13 after incubation under fluorescent light (500 lx for 24 h) are shown. Raw data are summarized in Table S1 in Supplementary Information.

We also subjected the photocatalyst coatings to antivirus assays for bacteriophage Qβ by Kitasato Research Center for Environmental Science and assays for bacteriophage M13 according to the procedures given in Japanese Industrial Standard JIS R1756 and those in Ref.^18^, respectively. Figure 5a shows the results for bacteriophage Qβ after fluorescent lamp irradiation (500 lx) for 4 h. The antivirus activities of the rutile- and anatase-based coatings were ≥ 5.0 and 3.4, respectively. The activity values were lowered to 1.6 (rutile) and 0.1 (anatase) for the control experiments without illumination (Fig. 5b). Those results show that the photo-renewing effect is very important to keep the high antivirus activities. On the other hand, the rutile-based photocatalyst keeps the significant antivirus activity even in the dark, reflecting that Cu_2_O remaining in the coating causes the antivirus effects because Cu_2_O has been known to inactivate bacteriophage Qβ^19,20^. In addition, both of the rutile- and anatase-based photocatalysts exhibited high inactivation effects on bacteriophage M13 (Fig. 5c); the antivirus activities were 3.6 and 5.0, respectively.

Antivirus effects on influenza A virus and feline calicivirus, which is often used as a surrogate for norovirus because of the similarity in terms of a capsid-enveloped structure, were also investigated in the dark by Kitasato Research Center for Environmental Science, according to the protocol of ISO 21702. The coatings were subjected to assay 18 days after the synthesis of the photocatalysts. We observed significant inactivation effects of the rutile- and anatase-based photocatalyst coatings after 24-h incubation, and the antivirus activities were > 3 for influenza A virus, and > 4 for feline calicivirus. The remaining Cu_2_O may also be effective for those viruses.

In previous studies, it has been shown that Cu_2_O inactivates influenza virus through denaturation of hemagglutinin^20^, which is a protein at the virus surface and binds to glycans with terminal sialic acid on host cells. We infer that Cu_2_O might also attack surface proteins of the novel coronaviruses, the bacteriophages Qβ and M13 and feline calicivirus. In the case of the novel coronavirus, it is known that there are spike proteins at the virus surface. The spike proteins bind ACE2, which is a receptor protein at the host cell surface, and lead to infection^21^. We therefore examined possible effects of the present photocatalyst on the binding ability of the spike proteins to human ACE2, in the following section.

### Effects on spike proteins of novel coronavirus

**Figure 6:**
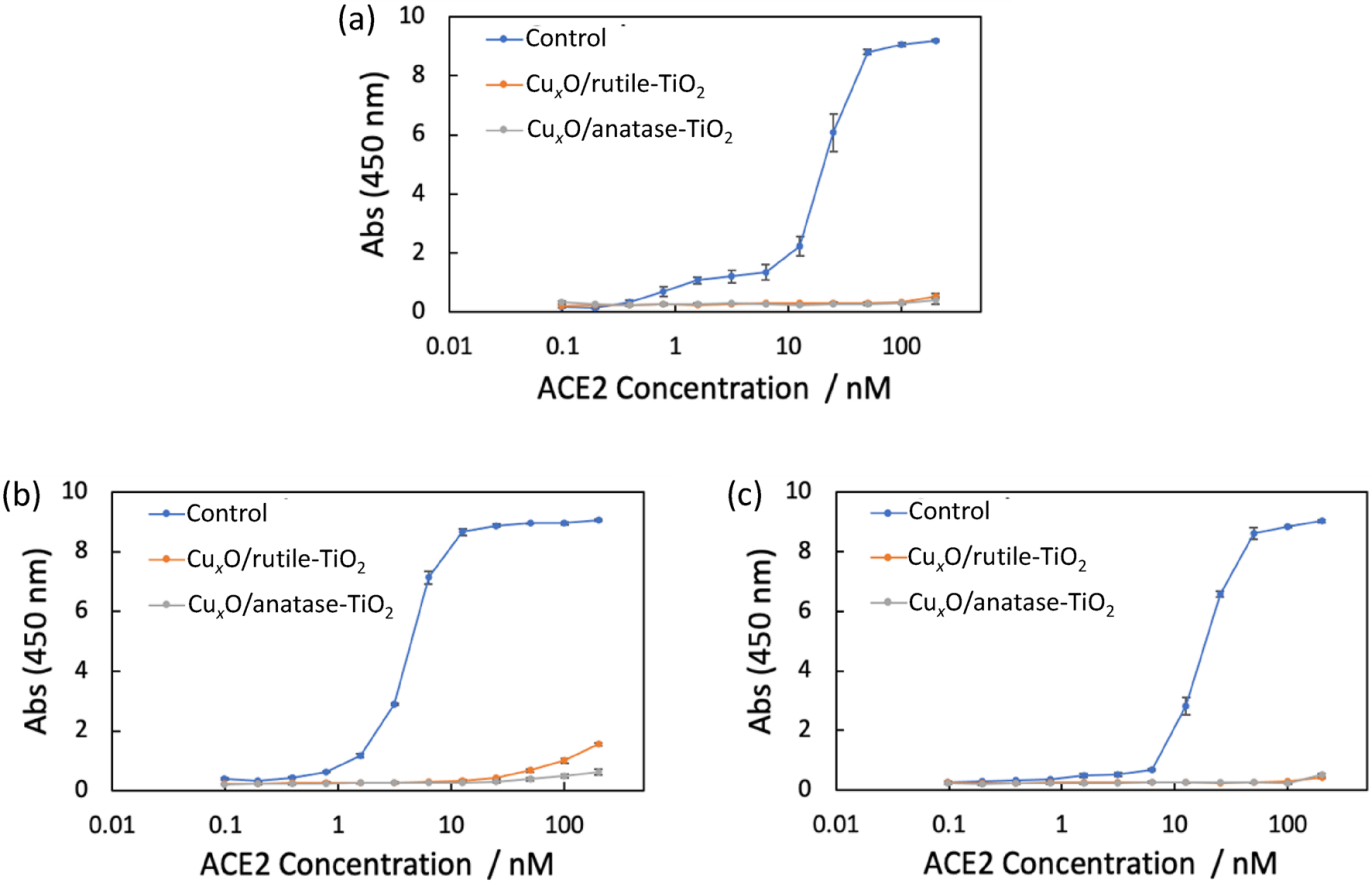
Denaturation effect of the rutile- and anatase-based photocatalysts on RBD domain of spike protein from (**a**) the original, (**b**) alpha and (**c**) delta variants of novel coronavirus. The binding activity of RBD to human ACE2 protein at various concentrations was assessed by ELISA (*n* = 3). Raw data are summarized in Table S2 in Supplementary Information.

We investigated if the photocatalysts containing Cu_2_O developed in this study denature the surface spike protein of novel coronavirus and thereby suppress its infectivity. To assess this, we prepared RBD of spike protein derived from the original, alpha and delta variants of novel coronavirus, and evaluated the denaturation effects of the photocatalysts on the protein. Since the binding of RBD to human ACE2 is an essential step in the infection^21^, the 5 µM recombinant RBD protein (70 µL) was mixed with the photocatalyst suspensions (70 µL) and left for 2 h at 4 °C and the binding activity toward ACE2 was examined by ELISA according to literature^22^. As shown in Fig. 6, the binding activity of RBD to ACE2 was significantly diminished by the incubation with the photocatalysts, indicating that the photocatalysts denatured the RBD protein. These results strongly support our conclusion that the antiviral effect of the photocatalyst on coronavirus relies, at least in part, on the protein denaturing activity. Importantly, RBD proteins derived from alpha and delta variants were also inactivated as was the original variant, indicating that the photocatalysis is effective to denature the spike protein regardless of mutation. It was suggested that the denaturation is due to disorder of electrostatic interaction in protein^20^. Further study to reveal the denaturing process of the proteins will provide a strategy to develop photocatalysts with even higher antiviral activity.

## Conclusions

Nanoscale Cu_*x*_O/TiO_2_ photocatalysts were prepared and applied to white and translucent latex paints. Cu_2_O clusters involved in the paint coating are gradually oxidized by ambient oxygen, while the oxidized clusters are re-reduced under > 380 nm light. The paint coating inactivated the original and alpha variant of novel coronavirus under fluorescent lamp irradiation. The photocatalysts denatured RDB of the original, alpha and delta variants of novel coronavirus and suppressed binding ability of their spike protein to human ACE2. The coating also inactivated influenza A virus, feline calicivirus, bacteriophage Qβ and bacteriophage M13. The photocatalytic paint coatings are expected to lower the risk of coronavirus infection via solid surfaces.

## Supplementary Information

Supplementary Information.

## Data Availability

All data generated or analysed during this study are included in this published article and its supplementary information file.
