# Supplementary material for: Inactivation and spike protein denaturation of novel coronavirus variants by CuxO/TiO2 nano-photocatalysts
Source: Sci Rep. 2023 Mar 10;13:4033. doi: 10.1038/s41598-023-30690-0 (PMC10000351; doi:10.1038/s41598-023-30690-0)
Supplement: Supplementary file 1 — Supplementary Information. [file 41598_2023_30690_MOESM1_ESM.docx]

Supporting Information

Inactivation and spike protein denaturation of novel coronavirus variants by Cu*_x_*O/TiO_2_ nano-photocatalysts

Tetsu Tatsuma^1,2,^*, Makoto Nakakido^1^, Takeshi Ichinohe^3,^*, Yoshinori Kuroiwa^2^,

Kengo Tomioka^4^, Chang Liu^4^, Nobuhiro Miyamae^4^, Tatsuya Onuki^1^, Kouhei Tsumoto^1,3,^*,

Kazuhito Hashimoto^1^ and Toru Wakihara^1^

^1^ *School of Engineering, The University of Tokyo, 7-3-1, Hongo, Bunkyo-ku, Tokyo 113-8656, Japan*

^2^ *Institute of Industrial Science, The University of Tokyo, 4-6-1 Komaba, Meguro-ku, Tokyo 153-8505, Japan*

^3^ *Institute of Medical Science, The University of Tokyo, 4-6-1 Shirokanedai, Minato-ku, Tokyo 108-8639, Japan*

^4^ *Nippon Paint Co., Ltd., 4-1-15 Minamishinagawa, Shinagawa-ku, Tokyo 140-8675, Japan*

Table S1. Raw data for Figures 4 and 5.

Table S2. Raw data for Figure 6.
